# Structure-Activity Relationships of the Antimicrobial Peptide Arasin 1 — And Mode of Action Studies of the N-Terminal, Proline-Rich Region

**DOI:** 10.1371/journal.pone.0053326

**Published:** 2013-01-11

**Authors:** Victoria S. Paulsen, Hans-Matti Blencke, Monica Benincasa, Tor Haug, Jacobus J. Eksteen, Olaf B. Styrvold, Marco Scocchi, Klara Stensvåg

**Affiliations:** 1 Norwegian College of Fishery Science, University of Tromsø, Tromsø, Norway; 2 Department of Life Sciences, University of Trieste, Trieste, Italy; 3 Department of Medical Biology, University of Tromsø, Tromsø, Norway; Aligarh Muslim University, India

## Abstract

Arasin 1 is a 37 amino acid long proline-rich antimicrobial peptide isolated from the spider crab, *Hyas araneus*. In this work the active region of arasin 1 was identified through structure-activity studies using different peptide fragments derived from the arasin 1 sequence. The pharmacophore was found to be located in the proline/arginine-rich NH_2_ terminus of the peptide and the fragment arasin 1(1–23) was almost equally active to the full length peptide. Arasin 1 and its active fragment arasin 1(1–23) were shown to be non-toxic to human red blood cells and arasin 1(1–23) was able to bind chitin, a component of fungal cell walls and the crustacean shell. The mode of action of the fully active N-terminal arasin 1(1–23) was explored through killing kinetic and membrane permeabilization studies. At the minimal inhibitory concentration (MIC), arasin 1(1–23) was not bactericidal and had no membrane disruptive effect. In contrast, at concentrations of 5×MIC and above it was bactericidal and interfered with membrane integrity. We conclude that arasin 1(1–23) has a different mode of action than lytic peptides, like cecropin P1. Thus, we suggest a dual mode of action for arasin 1(1–23) involving membrane disruption at peptide concentrations above MIC, and an alternative mechanism of action, possibly involving intracellular targets, at MIC.

## Introduction

Since the widespread resistance of bacterial pathogens against conventional antibiotics often makes antibiotic treatment inefficient, medical treatment of bacterial infections is today no longer a simple matter of prescribing antibiotic therapy. Therefore the interest in novel natural products with antimicrobial activity, as substitutes for the conventional antibiotics and new drug leads, is steadily increasing. Antimicrobial peptides (AMPs) are cationic and amphipathic molecules widely distributed amongst organisms of probably all taxonomic phyla and act as innate antibiotics. They are constitutively produced or synthesized after infection or injury, and exhibit desirable antimicrobial properties such as a rapid action and a broad activity spectrum [1], [2]. Approximately 1800 AMPs have been characterized [3], with the majority originating from natural sources. Substantial research remains in exploring these molecules' mechanism of action, and creating stable and easier-to-produce peptide analogs for medical applications.

Most AMPs inhibit microbial growth by permeabilizing cell membranes through a mechanism involving pore formation or other types of lytic effects allowing the efflux of essential ions and nutrients [4]–[6]. Examples of peptides believed to destroy membrane integrity include cecropins [7], magainins [8], [9] and many others [5]. Membrane active AMPs are not completely selective against microbial cells and are therefore potentially toxic to eukaryotic cells [10]. This is a drawback for these AMPs as candidates for new antimicrobial drugs, but they have a potential as anticancer drugs. Other AMPs showing higher selectivity for microbial cells are those which permeate bacteria without membrane disruption. These peptides do not target bacterial membranes, but instead bind to and inhibit intracellular molecules as their primary strategy of growth inhibition [11], [12].

Proline-rich AMPs (PR-AMPs) belong to the group of AMPs having intracellular targets [13]. They have a high content of proline and arginine residues and have shown to be predominately active against Gram-negative bacteria [14]–[18]. However, some PR-AMPs inhibit Gram-positive bacteria and exhibit broad-spectrum antifungal activity [19], [20]. PR-AMPs include the mammalian PR-39, Bac5 and Bac7 [15], [16], insect apidaecin [14], drosocin [21], pyrrhocoricin [17], shrimp penaeidins [22] and some other invertebrate peptides [13]. Studies on model membranes showed that such peptides were capable of binding to lipids or other membrane components, but they did not induce membrane lysis [23], [24]. Instead, PR-AMPs seem to interact with intracellular molecules involved in metabolic processes as evidenced by PR-39, which inhibits protein synthesis [7], and the insect peptides drosocin, pyrrhocoricin and apidaecin which inhibit the molecular chaperon DnaK [25], [26].

We have previously described the proline-rich AMP arasin 1 which was isolated from the hemocytes of the small spider crab *Hyas araneus* [27]. Arasin 1 is composed of 37 amino acids where proline and arginine account for a total of 32% and 19%, respectively. The overall structure of arasin 1 shows some similarity to penaedins [22]. It contains an N-terminal region dominated by proline and arginine residues, and a C-terminal region containing four cysteine residues forming two disulphide bridges. Arasin 1 shows activity against both Gram-positive and Gram-negative bacteria [27]. In contrast to membrane-active peptides like polymyxin B, cecropin P1 and B and lactoferricin B, arasin 1 generally displays minimal bactericidal concentration (MBC) values higher than minimum inhibitory concentration (MIC) values [27]. This pronounced difference between MBC and MIC suggests an alternative mechanism of action for arasin 1 compared to lytic AMPs.

In the present study we investigated the structure-activity relationship of arasin 1. A variety of synthetic arasin 1 fragments, covering the full-length peptide as well as the proline-rich N-terminus and the Cys-rich C-terminus were tested against a number of different microorganisms to evaluate the importance of the C- and N-terminal regions, and ultimately to develop a peptide, which is shorter and easier to synthesize. Moreover, killing kinetics, cell viability testing and membrane permeabilization assays were used to address the mechanism of action of the shortest fragment displaying full activity, arasin 1(1–23).

## Materials and Methods

### Synthetic peptides

Deletion peptides arasin 1(1–20) and (1–14) were provided by Thermo Fisher Scientific (Ulm, Germany), while a truncated version of PR-39, named PR-39(1–26) [28], and two linear thiol protected analogues of the full-length arasin 1 and its arasin 1(20–37) deletion peptide, named arasin 1-Acm and arasin 1(20–37)-Acm, respectively, were purchased from Biomol International (Exeter, UK). The thiol groups of these Acm-peptides were protected with acetamidomethyl groups to prevent any disulfide bridge formation. The full length (bicyclic) arasin 1 peptide and its arasin 1(1–23) deletion peptide were synthesized, cyclized and purified as previously described [27], [29]. Cecropin P1 and cecropin B [30], [31] were synthesized and purified as described by Kjuul *et al*. [32]. Arasin 1(3–23), (5–23), (7–23) and (9–23) were synthesized on a Tribute synthesizer (Protein Technologies Inc, Tucson, AZ, USA) following standard solid-phase Fmoc-chemistry protocols. After cleaving the completed peptides from the Rink amide resin (Novabiochem, Nottingham, UK), the crude peptides were purified on a C_18_ (100 Å; 5 µm; 20×250 mm) Inertsil ODS-3 column (GL Sciences Inc., Torrance, CA, USA) using a mixture of acetonitrile and water containing 0.1% TFA and a UV detection set at 214 nm. The purity of peptides were all greater than 95%, as determined by analytical RP-HPLC using a C_18_ (100 Å; 5 µm; 4.6×25 mm) SunFire column (Waters Associates, Millipore Corp., MA, USA) with the same conditions as described above. The correct molecular masses of the peptides were confirmed by MALDI-ToF mass spectrometry (MALDI micro MX, Waters Micromass Technologies, Münster, Germany). Mellitin B was purchased from Sigma-Aldrich (Haverhill, UK).

### Circular dichroism

The propensity of the peptides to assume a structure was probed by CD spectroscopy using a Jasco J-715 spectropolarimeter (Jasco, Tokyo, Japan), in 2-mm path-length quartz cells and with a peptide concentration of 20 µm, in either 10 mM sodium phosphate buffer (SPB) pH 7.0, in the absence or the presence of 10 mM SDS. All spectra are the mean of at least two trials, each with the accumulation of three scans. Ellipticity has been expressed per residue.

### Microorganisms

*Escherichia coli* (ATCC 25922), *E. coli* HB101, *Pseudomonas aeruginosa* (ATCC 27853), *Staphylococcus aureus* (ATCC 9144) and *Corynebacterium glutamicum* (ATCC 13032) were grown at 37°C in Mueller Hinton agar (MH; Difco Laboratories, Detroit, USA). The yeasts *Candida albicans* (ATCC 10231) and *Saccharomyces cerevisiae* spp and the mold *Botrytis cinerea* 101 [33] were cultivated on potato dextrose agar supplemented with 2% glucose at room temperature (RT).

### Antibacterial and antifungal assays

Determination of the MIC against bacteria was performed by continuous monitoring of bacterial growth using a Bioscreen C microbiology reader (Labsystems Oy, Helsinki, Finland). The synthetic peptides were tested for antibacterial and antifungal activity at final concentrations (prepared from serial two-fold dilutions) ranging from 0.2 µM to 100 µM. The test was performed in 100-well flat-bottomed honeycomb plates, in which 50 µl peptide solution was incubated with 50 µl of a suspension of an actively growing culture of bacteria diluted to a starting concentration of 2.5×10^5^ CFU/ml in Mueller Hinton Broth (MHB). The growth chamber was maintained at 37°C during the incubation period. The absorbance was measured by a turbidometric method with vertical light photometry and a wide band filter (420–580 nm). MIC was defined as the lowest concentration resulting in no visible bacterial growth after 24 h.

Fungal spores were dissolved in potato dextrose broth (Difco, Lawrence, KS, USA) and the cell concentration was determined and adjusted after counting in a Bürker chamber. *Botrytis cinerea* was filtered through a piece of cotton before counting. An aliquot of 50 µl of fungal spores (final concentration 2×10^5^ spores/ml) were inoculated in 96-well Nunclon™ microtitre plates (Nagle Nunc Int. Roskilde, Denmark) along with 50 µl of synthetic peptide dissolved in Milli-Q water. Cultures were grown in a dark moist chamber without shaking at 20°C for *S. cerevisiae* and *B. cinerea,* and at 37°C for *C. albicans*. Growth inhibition was determined microscopically after 48 h of incubation. Inhibitory concentrations (IC_50_) was defined as the lowest concentration tested which yielded less than 50% visible growth compared to the growth control (fungi). Each peptide was tested in parallels in three individual runs for the antimicrobial assays.

### Hemolytic assay

Arasin 1 and arasin 1(1–23) were tested for toxicity against eukaryotic cells using human red blood cells (RBC). Four milliliters of blood were collected from a healthy person into a polycarbonate tube containing heparin to a final concentration of 10 U/ml. The erythrocytes were pelleted by centrifugation at 450×g for 10 min and washed three times with phosphate-buffered saline (PBS; 320 mOsm, pH 7.4). The cell pellet was resuspended in 4 ml PBS and adjusted to a suspension giving 10% RBC. The test was performed in a 96 well U-shaped microtiter plate (Nunclon™ Surface, No. 163320; Nagle Nunc Int., Roskilde, Denmark), in which 50 µl peptide were incubated with 40 µl PBS and 10 µl RBC suspension. The synthetic peptides were tested at final concentrations ranging from 0.2 to 100 µM. After incubation in a shaker at 37°C for 1 h, the plate was centrifuged at 200×g for 5 min. The supernatants (60 µl) were carefully transferred to a new flat-bottomed polycarbonate microtiter plate (Nunc™ No. 269620, Nagle Nunc Int., Roskilde, Denmark), and absorbance was measured at 550 nm. Baseline hemolysis and 100% hemolysis were defined as the amount of hemoglobin released in the presence of PBS and 0.05% Triton X-100 (Sigma-Aldrich, Saint Louis, MO, USA), respectively. The EC_50_ value was taken as the mean concentration of peptide producing 50% hemolysis.

### Bactericidal assay

An actively growing culture of *E. coli* HB101 was diluted in 50% MHB to a starting concentration of 1×10^6^ CFU/ml, and incubated at 37°C with shaking for 4 h in the presence of arasin 1(1–23), cecropin P1 (positive control) or water (negative control). The final concentrations of peptide in suspension were 4 µM and 20 µM for arasin 1(1–23), and 1 µM for cecropin P1. During incubation, aliquots were removed at different time intervals, washed in buffered high salt solution (10 mM Na-phosphate, 400 mM NaCl, 10 mM MgCl_2_), diluted and plated on MH plates. The number of bacterial CFU was determined after approximately 48 h of incubation at 37°C.

### Cell viability assay

*E.coli* HB101 was transformed with the plasmid pCGLS-11 to enable constitutive bacterial luciferase expression from the *luxCDABE* operon. The resulting strain was used for cell viability assays as follows. An overnight culture in MH medium with ampicillin (100 µg/ml) was pelleted at 3000×g for 10 min and washed in MH medium before re-suspending the cells in 50% MH medium with a final OD_600_ of approximately 0.2. After 30 min of incubation at 30°C the cells were again adjusted to an OD_600_ of 0.2. An aliquot of 95 µl of bacterial solution was dispensed into each well of a black 96 well microtiter plate with transparent bottom (Falcon, Bredford, MA, USA). To achieve the indicated concentrations, a multi-channel pipette was used to add 5 µl of a two-fold dilution series of peptide solution in water to 8 wells simultaneously. The plate was loaded into a Perkin-Elmer Envision microtiter plate reader immediately after addition of the peptide (arasin 1(1–23), PR-39(1–26) or cecropin P1) and the measurement started 30 sec later because of handling events and 2 seconds of shaking inside the plate reader. Light production and growth (OD_595_) of the sensor bacteria was followed for 3 h under repeated shaking cycles before each measurement at 30°C.

### Membrane integrity assay I

*Escherichia coli* HB101 was transformed with the plasmids pCSS962 and the helper plasmid pGB3 to allow for constitutive expression of the eukaryotic luciferase gene *lucGR*. The resulting strain was isogenic to the other *E. coli* strains used. The membrane permeabilization assay used is a modification of the protocol described by Virta *et al.* [34]. The assay is based on *E. coli* cells, expressing a eukaryotic luciferase, depending on externally added D-luciferin as a substrate to emit light. At neutral pH the bacterial cytoplasmic membrane efficiently prevents uptake of external D-luciferin. However, disruption of the membrane by e.g. a membrane active AMP enhances D-luciferin influx and thereby induces bacterial light emission. Preparation of the cells and the assay plate was done as described for the viability assay above. However, chloramphenicol (30 µg/ml) was added in addition to ampicillin to the overnight culture, and D-luciferin sodium-salt (Biosynth, Staad, Switzerland) was added to the bacteria to final concentration of 1 mM. To allow shorter measurement intervals, only 8 wells were measured simultaneously. Arasin 1(1–23), PR-39(1–26) and cecropin P1 were tested for their membrane disruptive capacity.

### Membrane integrity assay II

The effect of arasin 1(1–23) and cecropin P1 on membrane permeabilization was also studied by measuring the percentage of propidium iodide (PI) positive cells by flow cytometry. Samples of 1×10^6^ cells/ml of *E. coli* HB101 were incubated in 50% MH with peptides at 37°C for different time span. After incubation, PI (Sigma Aldrich, Saint Louis, MO, USA) was added to the peptide-treated bacteria at a final concentration of 10 µg/ml. The cells were analyzed after 4 min of incubation with PI at 37°C in the flow cytometer as described [35].

### Chitin-binding assay

The ability of arasin 1(1–23) and arasin 1(20–37)-Acm to bind chitin was estimated using a modified protocol from Kawabata *et al*. [36]. Briefly, 50 µg of peptide was incubated with 40 mg chitin in 800 µl buffer (50 mM Tris-HCl, 0.1 M NaCl, pH 8.0). Sequentially chitin was incubated at RT with 800 µl buffer containing 0.1 M NaCl and 1 M NaCl, respectively, and finally at 95°C with 10% acetic acid. All incubations were performed for 10 min, and chitin was collected by centrifugation at 1000×g for 5 min between each step. Supernatants were subjected to RP-HPLC using a Sunfire™ Prep C_18_ column (Waters; 100 Å 5 µm; 10×250 mm, MA, USA), with a linear gradient from 8 to 60% acetonitrile and a flow of 3 ml/min over 60 min. Absorbance was monitored at 220 nm and the resulting chromatograms were compared.

## Results

### Antimicrobial activity of arasin 1 and truncated peptide fragments thereof

**Figure 1:**
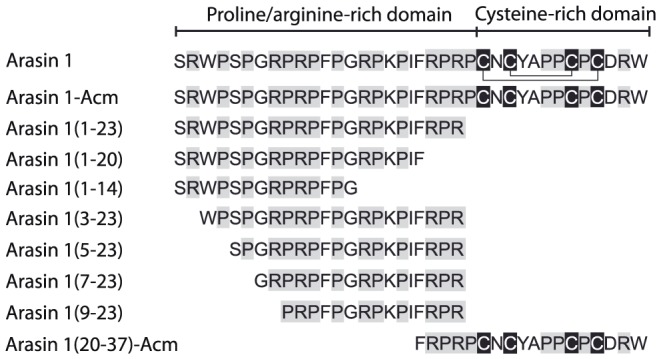
Overview of synthetic arasin 1 and its deletion peptides. Shaded letters indicate characteristic residues for arasin (Cys: black) and for proline-rich AMPs (Pro, Arg: grey). Acm = Acetamidomethyl. Predicted disulphide bridges are indicated for arasin 1 [27].

To address which part of arasin 1 is responsible for the antimicrobial activity, a series of truncated arasin 1 peptides were synthesized (Fig. 1), and their antimicrobial activity was determined and compared to arasin 1. The truncated peptides were derived from both the N- and the C-terminal part of arasin 1. In addition, a linear arasin 1 peptide (arasin 1-Acm) with protected thiol groups to prevent disulfide bridge formation was prepared. The results outlined in Table 1 represent the MIC and IC_50_ values of synthetic arasin 1 and peptide fragments derived from arasin 1 against bacteria and fungi respectively.

**Table 1:** Antibacterial and antifungal activity of synthetic arasin 1 and its derivatives^a^.

	MIC (µM)^b^					IC_50_ (µM)^c^		
Microorganisms	*E. coli* ATCC 25922	*E. coli* HB101	*P. aeruginosa* ATCC 27853	*C. glutamicum* ATCC 13032	*S. aureus* ATCC 9144	*S. cerevisiae* spp.	*C. albicans* ATCC 10231	*B. cinerea* 101
**Peptides**								
Arasin 1	3.1	6.25	6.3	0.8	50	6.3	12.5	12.5
Arasin 1-Acm	25–50	nd^d^	100	0.8	>100	12.5	25	12.5–50
Arasin 1(1–23)	6.3	3.1	12.5	0.8	>100	12.5	12.5	12.5
Arasin 1(1–20)	>100	25–50	>100	12.5–25	>100	25	50	25–50
Arasin 1(1–14)	>100	>100	>100	>100	>100	>100	>100	>100
Arasin 1(3–23)	25–50	12.5	100	3.1	>100	12.5	25	25
Arasin 1(5–23)	>100	100	>100	6.3	>100	12.5	25	25
Arasin 1(7–23)	50–100	25	50–100	3.1	>100	12.5	25	50
Arasin 1(9–23)	>100	100	>100	12.5	>100	25	50	50
Arasin 1(20–37)-Acm	>100	>100	>100	>100	>100	>100	>100	>100
Cecropin B	0.4–1.6	0.1	0.4	0.4–0.8	25	3.1	6.3–12.5	25
Cecropin P1	0.8–1.6	0.8–1.6	0.8–1.6	0.4–1.6	>100	6.3	12.5	50
PR39(1–26)	0.8–1.6	0.8–1.6	3.1–6.3	0.4	50	12.5	12.5	12.5

a The values outlined are the MIC and IC_50_ values for bacteria and fungi, respectively.

b MIC was defined as the lowest peptide concentration tested resulting in no visible bacterial growth. Bacteria were tested at 37°C in 50% MH at a concentration of 2.5×10^5^ cells/mL.

c IC_50_ was defined as the lowest concentration tested which yielded less than 50% visible growth compared to the growth control. Fungi were tested in and potato dextrose medium at 20°C, except for *C. albicans* that was tested at 37°C and at concentrations of 2.5×10^5^ cells/mL. Each data are representative of three individual experiments.

d nd: not determined.

The full length arasin 1 proved to be active not only against Gram-positive and Gram-negative bacteria, but also against the tested yeasts and mold at concentrations comparable to that of cecropins and PR-39(1–26). The linearization of the peptide (arasin 1-Acm) caused a strong decrease of antimicrobial activity especially in the case of Gram-negative bacteria. Arasin 1(1–23), corresponding to the N-terminal and proline-rich region of arasin 1, displayed activity comparable to the full length peptide. Further shortening from the C-terminus, resulted in a dramatic decrease or loss of antimicrobial activity, as demonstrated by arasin 1(1–20) and arasin 1(1–14), respectively. Differently, removal of 2–8 residues from the N-terminus of arasin 1(1–23) caused a drop of the antibacterial activity, in particular against Gram-negative bacteria. The C-terminal linear fragment arasin 1(20–37)-Acm, corresponding to the C-terminal and cysteine-rich region, displayed no antimicrobial activity up to 100 µM. MIC determinations showed identical MICs for arasin 1(1–24) and arasin 1(1–23) against all the strains from Table 1 (data not shown).

Overall these results indicate that the N-terminal proline-rich region of arasin 1 is responsible for the antibacterial and antifungal activities, and that a peptide consisting of the first 23 amino acids could be used in place of the complete peptide for further studies.

### Circular dichroism studies

**Figure 2:**
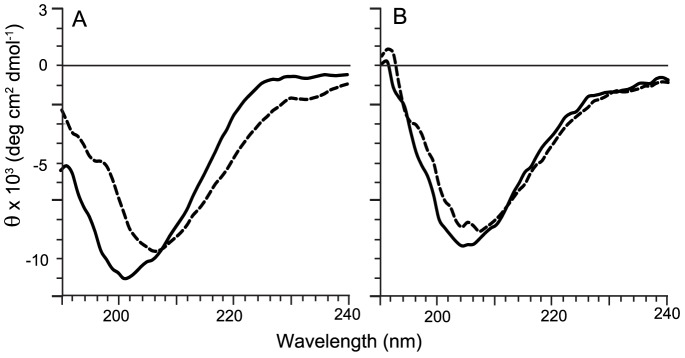
CD spectra of arasin 1(1–23) (A) and arasin 1 (B). Peptides were analyzed at a final concentration of 20 µM in 10 mM sodium phosphate buffer pH 7.0 (solid line) or with the addition of 10 mM SDS (dotted lines). Spectra are the smoothed means of at least two measurements, each composed of three different scans.

To strengthen the hypothesis that arasin 1(1–23) fragment may be used in place of the complete peptide, CD spectra for arasin 1 were recorded and compared to that of the fragment arasin 1(1–23). Spectra have been acquired in phosphate buffer or in presence of micellar SDS, used as a model of the negatively charged bacterial membranes. The two peptides showed a similar spectrum in aqueous solution, typical of that of unstructured peptides (Fig. 2). By the addition of the membrane-mimic SDS a small change in conformation was observed with the arasin 1(1–23). In this condition it adopted a spectrum more similar to that of the whole peptide which remained unchanged suggesting a more rigid structure (Fig. 2). As expected, cecropin P1 showed a CD spectrum with high α-helical content (data not shown).

### Killing kinetics of arasin 1(1–23)

**Figure 3:**
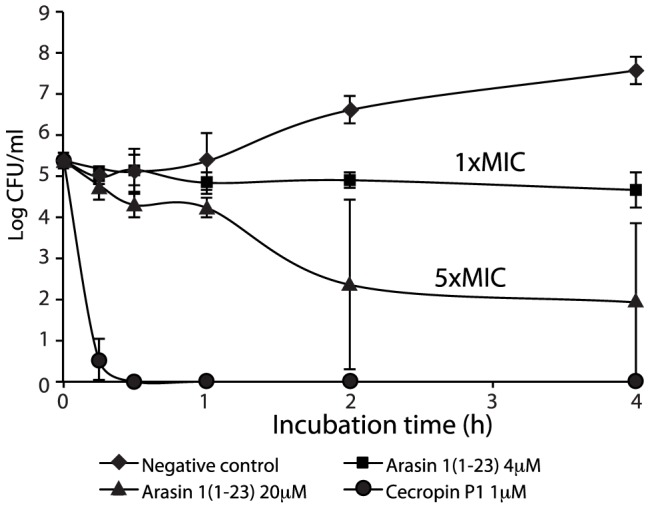
Killing kinetics of *E. coli* cells treated with arasin 1(1–23) or cecropin P1. Colony forming units (CFU) of *E. coli* HB101 subjected to peptide treatment or water (negative control) are shown. Sample aliquots were withdrawn and washed in high salt solution at the time points indicated and subsequently plated for colony counts. Data are expressed as the average number of colony forming cells ± S.D. for three independent experiments.

Killing kinetics of arasin 1(1–23) was studied against *E. coli* HB101. Exposure to MIC concentration (4 µM) of arasin 1(1–23) caused inhibition of bacterial growth without decreasing the number of viable cells (Fig. 3). A bactericidal effect was observed at 20 µM (5×MIC), causing a 1-log reduction of viable *E. coli* cells after 30 min. It is notable that the lytic peptide cecropin P1 (1 µM, MIC) killed all the bacteria in approximately 15 min, indicating a much higher rate of killing compared to that of arasin 1(1–23). After 2 and 4 h incubation with 20 µM peptide, unusual high standard deviations in the number of CFU/ml was observed. At this peptide concentration the bacteria seem to be very sensitive to small conditional variation associated with individual assay set-ups.

**Figure 4:**
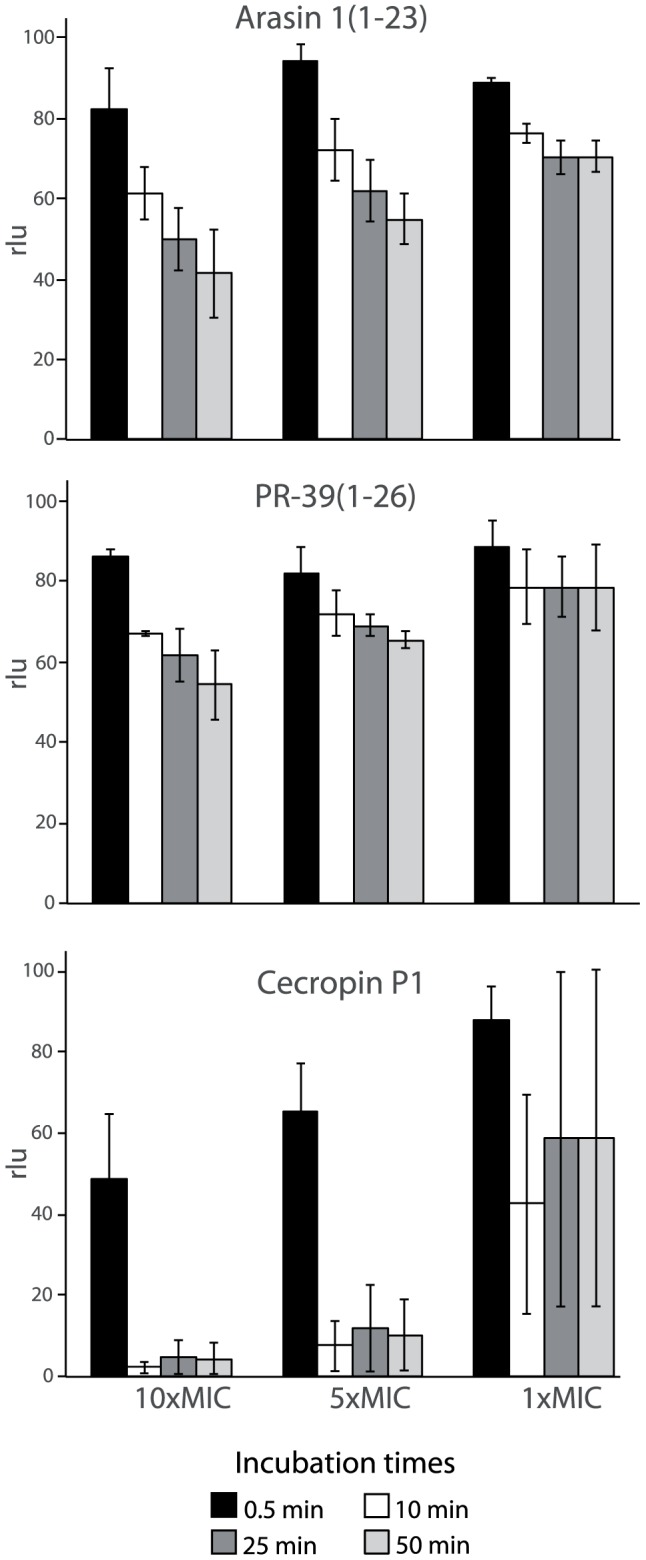
Effect of arasin 1(1–23), PR-39(1–26) and cecropin P1 on *E. coli* viability. Relative light units (rlu) produced by *E.coli* HB101, constitutively expressing the *luxCDABE* operon are shown, after AMP treatment at different concentrations. Light emission is shown relative to the untreated control for the 4 selected incubation times. The mean of three independent measurements is indicated ± S.D. MIC against *E. coli* HB101 is 4, 1 and 1 µM for arasin 1(1–23), PR-39(1–26) and cecropin P1, respectively.

Killing kinetics of peptide-treated bacteria were also evaluated using a luminescence-monitoring assay using an *E. coli* HB101 strain carrying the constitutively expressed *lux* operon [37]. Arasin 1(1–23)-treated bacteria maintained more than 60% luminescence after 10 min even at concentrations of 10-fold its MIC values (Fig. 4), indicating that most cells remained metabolically active. At MIC values (5 µM), the luminescence was more than 80% compared to untreated bacteria. Cells treated with PR-39(1–26) showed a similar decrease of cell viability (Fig. 4), whereas a dramatic drop in metabolic activity was observed after cecropin P1 treatment (MIC) with 60% reduction of light emitted within the first 10 min after addition of the peptide (Fig. 4). The number of bacteria (OD = 0.2) which was used in this assay was much higher than for the MIC and the former killing assay. This should be considered when comparing the results of the different assays. The killing caused by cecropin P1 occurred very rapidly when compared to arasin 1(1–23) which occurred more slowly. Full length arasin 1 was also tested, and the results were similar to those of arasin 1(1–23) (data not shown).

### Effect of arasin 1(1–23) on bacterial membranes

**Figure 5:**
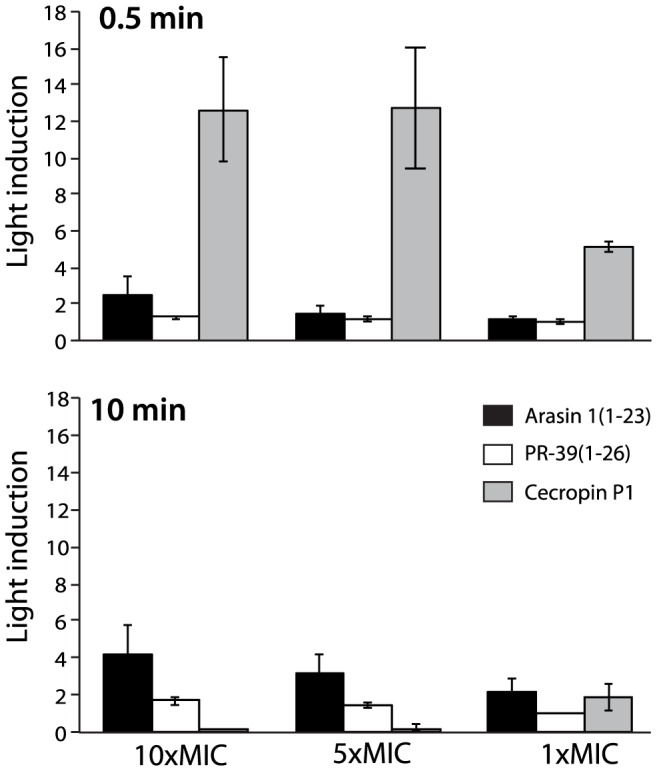
Effect of arasin 1(1–23), PR-39(1–26) and cecropin P1 on *E. coli* membrane integrity after 0.5 and 10 min incubation. Light induction of luciferase expressing *E. coli* HB101 after AMP treatment at different concentrations is shown. The data presented are relative to the untreated control for the two selected incubation times. The mean of three independent measurements is indicated ± S.D. Note that for the highly membrane active peptide cecropin P1 light emission for the highest concentrations already peaks before the first measurement. MIC against *E. coli* HB101 is 4, 1 and 1 µM for arasin 1(1–23), PR-39(1–26) and cecropin P1, respectively.

A real-time assay was used for *in vivo* detection of membrane permeation by arasin 1(1–23). Relative light production by *E.coli* HB101 constitutively expressing an insect luciferase requires externally added D-luciferin as its substrate. Light induction results from the increased passage of D-luciferin into the bacteria in response to decreased membrane integrity induced by the treatment with AMPs. Minimal amounts of light were emitted by cells treated with arasin 1(1–23) at a concentration around MIC value when compared to the effect due to cecropin P1 treatment (Fig. 5). At concentrations above MIC, arasin 1(1–23) seemed to be affecting membrane integrity as indicated by diagram doubling of light emission. Unlike cecropin P1, which permeabilized the membrane and lysed the cells within the first seconds, permeabilization caused by high concentrations of arasin 1(1–23) peaked after approximately 10 min. Also, unlike cecropin P1 light emission did not fall below the original level even after 50 min of incubation (results not shown), indicating viable cells. PR-39(1–26) shows a luminescence profile similar to that of arasin 1(1–23), in agreement with the results achieved from the viability assay.

**Figure 6:**
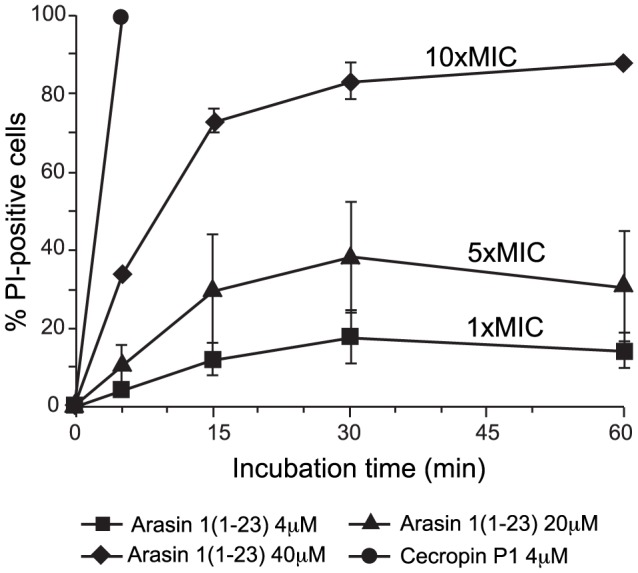
Bacterial membrane integrity after treatment with arasin 1(1–23) or cecropin P1. Percentage of fluorescent cells (PI-positive) measured by flow cytometry after incubation of *E. coli* HB101 cells with 4, 20, and 40 µM arasin 1(1–23) or 4 µM cecropin P1 is shown. The background level of permeabilized cells, obtained using untreated samples, was always lower than 3% and was subtracted to the corresponding peptide-treated sample. Data are expressed as the average of % PI-positive cells ± S.D. for four independent experiments.

To support the above results, we used flow cytometry to analyse the effect of arasin 1(1–23) on membrane integrity on the level of individual cells. Non-viable, permeabilized cells were marked using the fluorescent probe PI that incorporates and stains the nucleic acids only in cells with damaged membranes. Treatment of *E. coli* with arasin 1(1–23) at MIC amounts caused a low level of permeabilization (∼10%) (Fig. 6). The percentage of PI-positive cells increased (30%) when arasin 1(1–23) was used at 5×MIC, and a more rapid and marked effect was observed when *E. coli* was incubated with peptide at 10×MIC. In this case, after 15 min of treatment, approximately 75% of the cells became permeabilized (Fig. 5). By comparison, cecropin P1 induced damage of the membrane after only 5 min of incubation at MIC, showing a typical behaviour of lytic peptides and supporting a different mode of action for the two peptides. As shown in the cell viability assay (Fig. 2), a relatively high standard deviation at 20 µM peptide concentration has also been observed in this case.

### The antimicrobial N-terminal arasin 1(1–23) display chitin-binding properties

**Figure 7:**
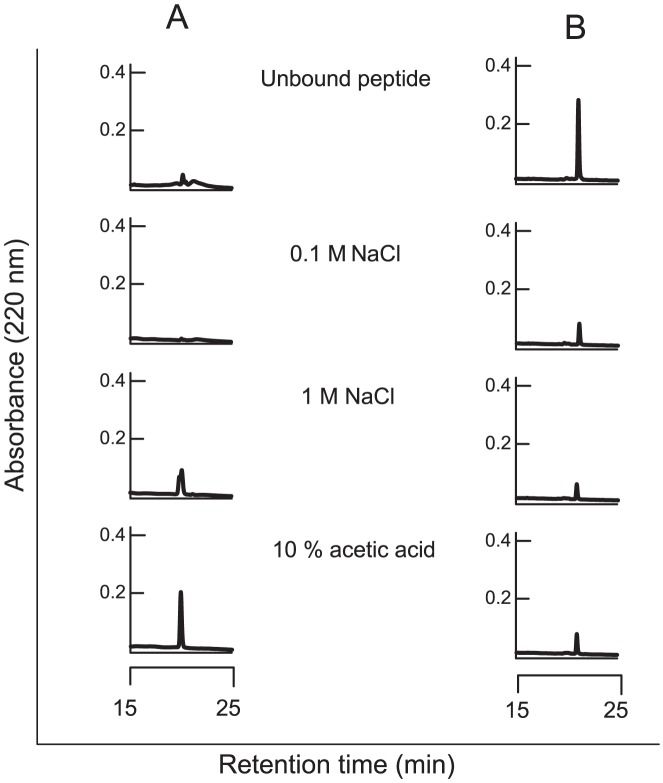
Chitin binding capacity of (A) arasin 1(1–23) and (B) arasin 1(20–37)-Acm. Fifty micrograms of peptides were incubated with 40 mg chitin, and subsequently washed with 0.1 M, 1 M NaCl, and hot (95°C) 10% acetic acid. The obtained supernatants were subjected to RP-HPLC on a C_18_ column. The resulting chromatograms show unbound material and peptides released from chitin by the different washing solutions, respectively.

Arasin 1(1–23) and the C-terminal fragment arasin 1(20–37)-Acm were tested for association to chitin. Arasin 1(1–23) remained bound to chitin after washing with salt and most peptide was not eluted before incubation with 10% acetic acid, indicating a strong binding of the peptide to chitin (Fig. 7). On the contrary, arasin 1(20–37)-Acm, did not bind tightly to chitin, as the bulk of the peptide was eluted by 0.1 M NaCl and only remnant peptide was found in the proceeding eluates.

### Hemolytic activity of arasin 1 and arasin 1(1–23)

Toxicity for eukaryotic cells was determined by testing arasin 1 and arasin 1(1–23) against human red blood cells (RBC). The peptides were incubated at final concentrations ranging from 0.2 to 100 µM in a 1% RBC suspension and EC_50_ values calculated after measuring the absorbance of released hemoglobin at 550 nm. Arasin 1 and arasin 1(1–23) showed no detectable hemolytic effect up to 100 µM concentration, compared to the 100% hemolysis and zero control. In contrast, the hemolytic mellitin B and cecropin B showed EC_50_ values of 2 and 40 µM, respectively.

## Discussion

Arasin 1 which is a PR-AMP [27], was active against the whole panel of test strains used in this study (Table 1). Thus, it seems to have an activity spectrum which is broader than the spectra of some other known PR-AMPs. For example the penaeidins, a proline-rich peptide family with a unique di-domain structure similar to arasin 1, are active predominantly against Gram-positive bacteria and fungi [19], [20]. In contrast, the activity of the mammalian proline-rich peptides Bac7 and Bac5, and the insect peptides pyrrhocoricin, drosocin and apidaecin, are mainly directed against Gram-negative bacteria [14], [15], [17], [18].

Antimicrobial activity of the N-terminal region of arasin 1, arasin 1(1–23), was comparable to that of the entire peptide (Table 1). These molecules resemble each other also in their secondary structure. This indicates that the antimicrobial pharmacophore resides within the N-terminal region of arasin 1 and that the C-terminal segment is not requisite for antimicrobial activity. In addition dichroic spectra of both the peptides are compatible with random coil and the shape of the spectra are not significantly modified in the presence of liposomes or micelles indicating that interaction with membranes does not markedly modify their structure. Arasin 1(1–37)-Acm (with blocked cysteines) was synthesized to explore the role of the disulphide bridges for antimicrobial activity. This linear peptide expressed lower activity against Gram-negative bacteria compared to the original peptide. Except for this, the antimicrobial activity of the linear peptide was comparable to the activity of arasin 1 and arasin 1(1–23). There is a slight possibility that the introduced Acm-modification affects the activity of the synthetic peptide, by interfering with the uptake mechanism through the outer membrane of the Gram-negative bacteria. In addition, activity testing of the linear C-terminal arasin 1(20–37)-Acm, revealed no antimicrobial activity of this peptide fragment. Altogether, these results support the conclusion that the cysteine-containing C-terminus is not essential for antimicrobial activity. On the other hand, however, the C-terminal residues may have different properties other than antibacterial activity. AMPs as host defense peptides have several roles in host immunity, taking part (directly or indirectly) in combating infections and/or wound healing [38]. Thus, the C-terminus or the entire peptide could be important, if arasin 1 has such properties. Alternatively, the disulfide bonds and the loop structure of the C-terminus may offer enhanced stability to the peptide and consequently reduced sensitivity to proteolytic cleavage. Also other PR-AMPs like Bac5, Bac7 and PR-39 have been subjected to structure-activity studies and in accordance with arasin 1, the N-terminus has proven to be the active region [20], [28], [39]–[41]. Like arasin 1, they all have a highly cationic N-terminal region due to high contents of arginine residues, which is probably important for attracting these peptides towards target bacteria. However, the proline-rich region of a class 4 (Pen4-1 from *Litopenaeus setifierus*) but not a class 3 penaeidin isoform (Pen3-4 from *L. vannamei*) expressed antibacterial activity with the same target species spectrum as the mother peptide. The pro-rich region of the latter peptide diverge from the bulk PR-AMPs by proving to be antimicrobial inactive [20]. Still, in accordance with the findings of arasin 1, the proline/arginine-rich N-terminus seems to be important for antimicrobial activity of most PR-AMPs.

N- and C- terminal shortening of arasin 1 (1–23) revealed the importance of the C-terminal residues RPR, but also the N-terminal residues or a length of 23 amino acids. Antimicrobial activity of arasin 1(1–24) proved to be identical to that of arasin 1(1–23) against all strains displayed in Table 1, signifying that an additional proline residue does not seem to affect, and thus have no role in antimicrobial activity.

The arasin 1 fragments (1–21) and (1–22) however, need yet to be evaluated. There is the possibility that one of these fragments shows antimicrobial activity equal to or higher than arasin 1(1–23). The shortening involves the removal of a hydrophobic proline or a cationic arginine. The importance of a Pro residue in specific positions for antibacterial activity, was proven by amino acids substitution and mutation studies in some insect PR-AMPs [42], [43].

On the other hand, the number of cationic arginines (positive charge), rather than the percentage of prolines present in the sequence, seems to affect the antimicrobial activity of AMPs. This is true for the PR-AMPs Bac5, Bac7 and PR-39 fragments, for which a minimum charge of +8 was required for full activity maintenance [41], [44]–[46]. A charge of +7 was necessary for a maximum antimicrobial effect of the arasin 1 fragments. Substitution of all arginine residues with lysines in Bac5(1–23) however, caused the antibacterial activity to drop drastically although the net charge was unchanged [40]. This indicates that charge is not the only requisite for antimicrobial activity. The importance of arginines and other residues in arasin 1 peptides need to be assessed, by replacing specific amino acids for others and conducting alanine or lysine scans.

In contrast to the results from *E. coli* and *P. aeruginosa*, the C-terminal part of arasin 1(1–23) alone arasin 1(7–23) and 1(9–23) inhibits *C. glutamicum* and all the fungi. At the same time this antibacterial and antifungal activity is strictly dependent on a fragment between aa 13–23. This indicates a somewhat different mode of action for Gram- negative and Gram-positive bacteria, respectively. Likewise the mode of action against fungi is likely different. It is tempting to speculate that the N-terminal 5–10 amino acids might be involved in arasin 1(1–23) transfer across the outer membrane while the C-terminal part might bind the molecular target which is responsible for growth inhibition at low concentrations. The antimicrobial assays were all performed in MH or potato dextrose medium at a final concentration of 50% strength for bacteria and fungi, respectively. This was done because the arasin 1 fragments were less active (MIC>32 µM) when medium strength of 100% were used. This means that the peptides are very sensitive to medium composition, and perhaps also salinity and/or ionic strength. The mammalian PR-AMPs PR-39(1–26) and Bac7(1–16) did not change the antimicrobial activity against *E. coli* by varying the MH concentration (results not shown). In some studies, the use of diluted or poor medium instead of rich medium in MIC and mode of action studies is claimed to increase the sensitivity of the assays [18], [47]. The reasons of the medium-dependence have not been further evaluated, and 50% MH was used as the standard medium for the mode of action studies.

The fungal cell wall is a complex structure composed of chitin, glucans and other polymers. The tight binding of the antifungal arasin 1(1–23) to chitin may imply that the peptide, like other chitin binding antimicrobial proteins and peptides, interferes with fungal growth by associating to nascent chitin in the fungal cell wall [48]–[52]. The strong association with chitin could however be the initial interaction for a further translocation of the peptide to the cytoplasmic membrane, and ultimately into the cytoplasm. The chitin binding properties may also be due to arasin 1 being involved in wound healing in the host organism, the spider crab. Chitin from the exoskeleton of the crustacean may be exposed at the site of a lesion and act as an attractant for arasin 1. The latter function has been proposed for chitin binding peptides from other arthropods e.g. penaeidins from the shrimp *Penaeus vannamei* [53], tachystatins from the horseshoe crab *Tachypleus tridentatus* [52], and hyastatin isolated from the spider crab *H. araneus* [54].

The bactericidal kinetics of arasin 1(1–23) differed fundamentally from that of cecropin P1. In contrast to cecropin P1, the killing profile of arasin 1(1–23) was similar to PR-39(1–26), another proline-rich AMP. While cecropin P1 proved to be a bactericidal agent - killing bacteria rapidly at MIC, arasin 1(1–23) and PR-39(1–26) conducted growth inhibition rather than to kill bacteria at their MICs. However, a bactericidal effect was observed for arasin 1(1–23) and PR-39(1–26) at concentrations several times above the MIC, but occurred at a much slower rate compared to the effect of cecropin P1. This was observed by using two different assays: killing kinetics and bioluminescence. Note that, these two methods are not directly comparable, regarding the order of magnitude. Detectable and reliable relative light unit (rlu) levels for the bioluminescence based assay, requires a higher concentration of bacteria compared to the colony count assay. Regardless of the method used, the results indicate that arasin 1(1–23) affects and kill bacteria by a different mode of action than cecropin P1 and seem to be more similar to PR-39(1–26).

Cecropin P1 has been shown to act upon the bacterial cell membrane and to induce lysis through pore formation [7], [55], as was confirmed in this study. The treatment of bacteria by the peptide made *E. coli* fluorescent and luciferase expressing *E. coli* started to emit light due to rapid diffusion of PI or D-luciferin, respectively, from the growth medium into the cytoplasm. Since PI or D-luciferin influx is obstructed by an intact cytoplasmic membrane, these results indicate the formation of pore or the complete lysis of the cell. While addition of cecropin P1 to the sensor strains instantly resulted in intensive fluorescence or light emission at MIC, the presence of arasin 1(1–23) and PR-39(1–26) at MIC did not induce an intensively enhanced signal. The increased fluorescence and light emission after addition of arasin 1(1–23) above MIC, indicates a decrease in membrane integrity at high peptide concentrations. This corresponds with the results obtained for PR-39(1–26). Even though the PR-AMPs rendered membranes leaky above their MICs, cecropin PI on the other hand showed a much higher rate of action leading to more PI and D-luciferin influx.

The outcome of the membrane permeabilization studies are in accordance with the observed difference in killing kinetics for arasin 1(1–23) and cecropin P1, and the results by Stensvag *et al.* [27] showing that arasin 1, in contrast to membrane-active peptides (polymyxin B, cecropin P1, cecropin B and lactoferricin B) has lower MIC than MBC. Therefore we propose that the mode of antimicrobial action of arasin 1 differs from that of cecropin P1. Because arasin 1(1–23) is showing a similar behavior to PR-39(1–26), we speculate that arasin 1 acts in a similar manner as this mammalian peptide. Studies using model membranes demonstrated no occurrence of channel formation by PR-39 [24]. Neither did its N-terminal fragment PR-39(1–26) lyse bacterial cells through pore-formation [28]. In addition, it has been suggested from isotope incorporation studies that PR-39 kills bacteria by inhibition of both protein and DNA synthesis [7]. The PR-39(1–26) and arasin 1(1–23) killing profiles are quite similar, therefore it is tempting to speculate that the mode of action for arasin 1(1–23) also involves intracellular target molecules at low, near MIC concentrations. It has been shown that intracellular macromolecules are the targets of a number of other proline-rich AMPs [25] [26]. A stereospecific binding to a putative intracellular interaction partner DnaK and uptake into *E.coli* was demonstrated for fully active Bac7(1–35) [35], [56]. In addition, Podda *et al*. [35] found that Bac7 fragments at concentrations several times higher than the MIC, acted also by a second mechanism based on a non-stereo selective, membranolytic activity. It is interesting to note that also arasin 1(1–23), at concentrations well above MIC values showed a similar lytic activity, as did PR-39(1–26).

The high standard deviation values only observed at 20 µM (5×MIC) for both the viability assay (Fig. 3) and the membrane integrity assay (Fig. 6) indicate that activity around this concentration was very sensitive to individual assay conditions. This suggests that at approximately 20 µM, arasin 1(1–23) switches from being membrane non-disruptive to using in addition a membrane disruptive mechanism and becoming more bactericidal. Notably, cecropin P1 which has MBC values against Gram-negative bacteria at 1–2 µM [27], display high standard deviations around its MIC/MBC in the viability assay (Fig. 4).

Arasin 1 and arasin 1(1–23) are not toxic to human red blood cells even at high (100 µM) concentrations. This is in contrast to mellitin B, which caused hemoglobin release at 2 µM and cecropin B causing 50% hemolysis at 40 µM. The absence of hemolytic activity is important when considering the AMPs as candidates for clinical or food additive purposes.

In this study, the antibacterial and antifungal activity of arasin 1 was addressed to the N-terminal fragment arasin 1(1–23). The arasin 1 pharmacophore should be further elucidated to reveal scaffold amino acids responsible for antimicrobial activity. Therefore, extended structure-activity studies, including alterations of the peptide sequence derived from the N-terminus of arasin 1, should be undertaken. Arasin 1(1–23) show a mechanism of action with most elements resembling that of other PR-AMPs, while at the same time exhibiting some characteristics such as the medium-dependent activity and a lytic action when used at higher concentrations (>20 µM). Overall, these results imply a membrane non-disruptive peptide uptake mechanism for arasin 1(1–23), which makes us speculate that the antibacterial strategy involves intracellular target molecules. Our further focus will be on the internalization mechanism of arasin 1 active fragments, using MIC and sub-MIC concentrations of the peptide, and further on search for molecular interaction partners for these peptides. This will provide valuable information considering peptidomics and future use of proline-rich peptides in drug design.
